# Infection Prevention and Control at Lira University Hospital, Uganda: More Needs to Be Done

**DOI:** 10.3390/tropicalmed6020069

**Published:** 2021-05-01

**Authors:** Marc Sam Opollo, Tom Charles Otim, Walter Kizito, Pruthu Thekkur, Ajay M. V. Kumar, Freddy Eric Kitutu, Rogers Kisame, Maria Zolfo

**Affiliations:** 1 Department of Public Health, Faculty of Health Sciences, Lira University, 1035 Lira, Uganda; otimtomc@gmail.com; 2 Médecins Sans Frontières, MSF-B, 1050 Brussels, Belgium; Walter.Kizito@brussels.msf.org; 3 International Union against Tuberculosis and Lung Disease (The Union), 75006 Paris, France; Pruthu.TK@theunion.org (P.T.); akumar@theunion.org (A.M.V.K.); 4 The Union South-East Asia Office, New Delhi 110016, India; 5 Yenepoya Medical College, Yenepoya (Deemed to be University), Mangaluru 575018, India; 6 Sustainable Pharmaceutical Systems (SPS) Unit, Pharmacy Department, Makerere University School of Health Sciences, 10217 Kampala, Uganda; kitutufred@gmail.com; 7 Infectious Diseases Institute, Makerere University, 10217 Kampala, Uganda; kisamerogers@yahoo.com; 8 Institute of Tropical Medicine, 2000 Antwerp, Belgium; mzolfo@itg.be

**Keywords:** core components of infection prevention and control, healthcare-associated infections, operational research, SORT IT (Structured Operational Research and Training Initiative), low-income and middle-income countries

## Abstract

Globally, 5–15% of hospitalized patients acquire infections (often caused by antimicrobial-resistant microbes) due to inadequate infection prevention and control (IPC) measures. We used the World Health Organization’s (WHO) ‘Infection Prevention and Control Assessment Framework’ (IPCAF) tool to assess the IPC compliance at Lira University hospital (LUH), a teaching hospital in Uganda. We also characterized challenges in completing the tool. This was a hospital-based, cross-sectional study conducted in November 2020. The IPC focal person at LUH completed the WHO IPCAF tool. Responses were validated, scored, and interpreted per WHO guidelines. The overall IPC compliance score at LUH was 225/800 (28.5%), implying a basic IPC compliance level. There was no IPC committee, no IPC team, and no budgets. Training was rarely or never conducted. There was no surveillance system and no monitoring/audit of IPC activities. Bed capacity, water, electricity, and disposal of hospital waste were adequate. Disposables and personal protective equipment were not available in appropriate quantities. Major challenges in completing the IPCAF tool were related to the detailed questions requiring repeated consultation with other hospital stakeholders and the long time it took to complete the tool. IPC compliance at LUH was not optimal. The gaps identified need to be addressed urgently.

## 1. Introduction

Healthcare-associated infections (HCAIs) are those infections occurring in a patient during the process of care in a hospital or other healthcare facility, which was not present or incubating at the time of admission. This includes infections acquired in the hospital, but appearing after discharge [1]. HCAIs also include occupational infections that occur among the healthcare workers. Globally, 5–15% of hospitalized patients suffer from HCAIs and this is primarily due to poor infection prevention and control (IPC) practices in the hospitals. In low-income and middle-income countries (LMICs), the frequency of HCAIs is estimated to be more than double compared to high-income countries. A total of 16% of patients acquire HCAI at any given time in LMICs as compared to 5–7% of patients in high-income countries, reflecting the differences in compliance to IPC practices [2]. HCAIs result in increased duration of hospitalization, morbidity, mortality, and high costs for patients, families, and health systems. The current COVID-19 pandemic has emphasized the need for high levels of IPC in healthcare facilities and communities. Although not representative, data reported from many countries indicate that around 14–35% of COVID-19 cases reported to WHO are among health workers. Hence, implementation of IPC is crucial in healthcare facilities [3].

HCAIs are often caused by drug-resistant microbes present in water, food, and the environment of a hospital setting and transmitted by both patients and health workers due to poor IPC practices [4]. Decades of excessive antimicrobial use in humans and animals have led to the emergence of antimicrobial resistance (AMR) [5]. Every infection prevented equates to one less instance of antibiotic use. Thus, IPC is one of the main pillars of the global framework to reduce antimicrobial resistance (AMR). AMR is a growing threat to public health, reducing the ability of antimicrobial agents to control infections and causing a high burden of morbidity, mortality, and financial losses for the healthcare systems. Several studies have indicated that appropriate hand hygiene practices significantly reduce the risk of HCAIs, while poor hand hygiene along with overcrowded and understaffed health facilities are associated with steep increases in their prevalence [6,7,8]. Clearly, a high proportion of HCAIs are preventable through simple and effective IPC measures, such as hand hygiene and use of personal, protective equipment [9,10,11,12]. Health workers’ knowledge and understanding, accompanied by the correct attitude toward IPC, is important for its effective implementation [13].

In Uganda, infectious diseases account for 18% of all hospital deaths and 37% of hospital admissions [14]. To combat AMR, Uganda has developed policies and guidelines including a “National Action Plan 2018–2023” against AMR to prevent, slow down, and control the spread of resistant microorganisms [14]. As a part of this plan, IPC guidelines are expected to be implemented throughout the country, led by an IPC committee at a national level and in every tertiary health facility.

In 2019, the World Health Organization (WHO) launched a global survey to encourage facility-level assessments of IPC and hand hygiene activities in order to gather a situational analysis on the level of IPC program compliance around the world [15]. Healthcare facilities are encouraged to complete an IPC Assessment Framework (IPCAF), a systematic IPC self-assessment tool. The IPCAF detects relevant problems and shortcomings needing improvements in order to meet international standards and requirements. Through repeated administration, this self-assessment tool, based on eight IPC core components, can document progress overtime and facilitate IPC progress [16].

To date, there is no information on the level of IPC compliance, strengths, and weaknesses in the implementation of IPC and the IPC assessment has never been conducted at Lira University Hospital (LUH), which is a teaching hospital that started in 2019 with the support of the African Development Bank.

The aim of this study was to assess the IPC compliance (i.e., adherence to WHO guidelines, training/education, availability of materials and personnel for IPC) at LUH, using the WHO IPCAF, specifically, (i) assessing the baseline level of IPC compliance, (ii) identifying strengths and gaps in implementing IPC activities at the facility, and (iii) reporting the challenges encountered by the IPC focal person in completing the IPCAF tool.

## 2. Materials and Methods

### 2.1. Study Design

This was a hospital-based cross-sectional study.

### 2.2. Setting

**Figure 1:**
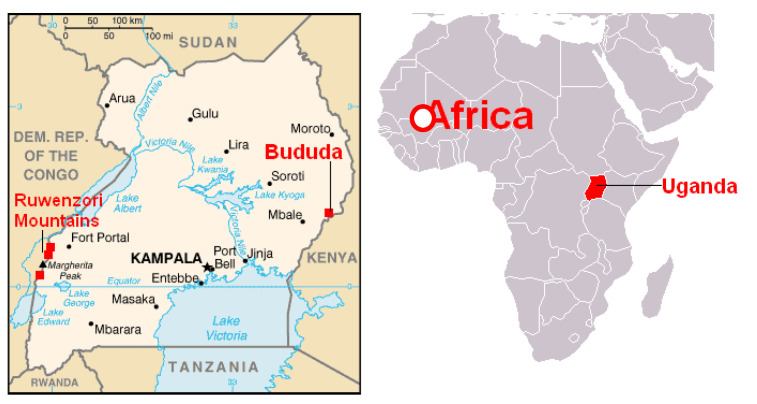
Map of Uganda showing position of Lira municipality.

Uganda is a land-locked East African country with a population of 41.6 million in June 2020 (Uganda Bureau of Statistics). It borders South Sudan to the north, Kenya to the east, Tanzania and Rwanda to the south, and the Democratic Republic of Congo to the west. Uganda has 13 health regions with five national referral hospitals, 13 regional referral hospitals, 163 general hospitals, and a network of primary health centres. The mandate of implementing healthcare delivery in Uganda lies with the Ministry of Health (MoH), and the health service providers include public (government, including uniformed services and university hospitals), private not-for-profit (faith-based), private for-profit, and nongovernmental organisations (NGOs). Lira municipality, located in the mid-northern region of the country, is around 340 km from Kampala, the capital city of Uganda (Figure 1).

### 2.3. Study Site

The study was conducted at LUH, which is a teaching hospital for the medical students in the University. The hospital has a capacity of 110 beds and is staffed by 97 healthcare workers. The hospital has several specialist departments including obstetrics, gynecology, pediatrics, surgery, medicine, and pathology and provides comprehensive health services to the general public.

### 2.4. Data Collection and Validation

Data were collected using the self-administered WHO IPCAF tool (supplementary Table S1). The tool has eight core components: (i) IPC programme, (ii) IPC guidelines, (iii) IPC education and training, (iv) HCAI surveillance, (v) multimodal strategies for implementation of IPC interventions, (vi) monitoring/audit of IPC practices and feedback, (vii) workload, staffing, and bed occupancy, and (viii) built environment, materials, and equipment for IPC at the facility level [17].

The clinical supervisor of the hospital acted as an IPC focal person and completed the IPCAF tool. Whenever in doubt, the clinical supervisor contacted the most informed people from each of the six departments mentioned above and filled in the information. The principal investigator cross-validated the information filled in by the clinical supervisor. The IPC focal person also reported on the challenges encountered in completing the IPCAF data tool and these were noted. The data were collected at LUH in November 2020.

### 2.5. Data Analysis and Statistics

The responses were assessed and scored as per the WHO guidelines. Every component of the IPCAF tool contributed to a score of 100 and, thus, the maximum score that could be obtained was 800. Based on the overall score, the IPC compliance was categorized as inadequate (0–200), basic (201–400), intermediate (401–600), and advanced (601–800) (see Table 1). Based on the responses in each component, a score was obtained and a percentage score was calculated (score obtained divided by the maximum component score multiplied by 100). Compliance against each component was graded based on the component score percentage: (i) inadequate (0–25%), (ii) basic (25.1–50%), (iii) intermediate (50.1–75%), and (iv) advanced (75.1–100%). IPC sub-components attaining the maximum score were considered “strengths” and components with zero or less-than-full scores were considered to be “gaps.” The strengths and gaps are summarized in the tables segregated by each core component. The challenges encountered by the IPC focal person in filling the IPCAF tool were summarized as a narrative.

## 3. Results

### 3.1. Baseline Level of IPC Compliance at LUH

All the six departments of the Lira University Hospital were assessed. The overall IPC compliance score was 220/800 (27.5%), which equates to a ‘basic’ level of compliance. For the individual components of IPCAF, scores ranged from 0% to 77.5%. The scores for most of the IPCAF components ranged from 0–25%, which is considered an ‘inadequate’ level of compliance. The best compliance was observed in the ‘Built Environment, materials, and equipment’ component (Table 2). The full details of the responses to the IPCAF questionnaire are shown in Supplementary Table S1.

### 3.2. Strengths and Gaps in Implementing IPC Activities at LUH

The strengths and gaps related to the different components of the IPC framework are detailed in Table 3, Table 4 and Table 5. Consistent with the scores, there were gaps in all the components, but the most numerous were observed under the IPC programme, IPC guidelines, and monitoring/audit components of the IPCAF tool.

Table 3 details the strengths and gaps in the IPC programme, guidelines, education, and training. There was no IPC programme in the hospital, no IPC team with full-time dedicated professionals trained in IPC and no IPC committee, comprised of multi-disciplinary staff members. Even the focal person identified for IPC did not have dedicated time to focus on IPC. No budgets were allocated for IPC activities in the hospital. Barring guidelines on hand hygiene, no other WHO-recommended guidelines were present in the facility. While the expertise to conduct the training was present, training programmes were rarely carried out for healthcare workers and no trainings were ever conducted for administrative and managerial staff, patients, or their family members.

Table 4 details the strengths and gaps in surveillance and monitoring. None of the multimodal strategies recommended to promote IPC (system change, education and training, monitoring and feedback, communication and reminders, safety climate, and culture change) were used. While personnel responsible for surveillance were present and trained, no surveillance activities were conducted. Surveillance methods were not defined. Case definitions were not as per international standards and there were no processes to monitor data quality.

Table 5 details the strengths and gaps in the ‘built environment, materials, and equipment’ and ‘workload, staffing, and bed occupancy’ components. Bed occupancy was maintained at one patient per bed and there was a system in place to assess and respond when adequate bed capacity was exceeded. All the essential services such as safe water for drinking and other purposes, electricity supply, sanitation, and disposal of hospital waste were available for the facility. Gaps in the built environment component included inadequate documentation of hygiene practices, presence of non-functional toilets, latrines, and waste management equipment. Disposable items and PPE equipment were not available in adequate quantities at all times.

### 3.3. Challenges Encountered by the IPC Focal Person in Completing the IPCAF Tool

The major challenge in filling the IPCAF tool was lack of knowledge on part of the IPC focal person about the contents of the questionnaire. It was observed that the tool is lengthy with many questions in different areas, which required consultation with several departmental heads. Due to the length, it took a long time to fill the questionnaire (1 to 3 h) and needed focused time away from the demands of routine work, which was perceived to be a major challenge.

## 4. Discussion

Our findings indicate that the IPC compliance at LUH was ‘basic’, meaning many aspects of the IPC core components were either not in place or not adequately implemented. Out of eight components on IPC compliance, five were graded ‘inadequate.’ Another related to IPC education and training was graded ‘basic’. The one related to bed occupancy, staffing, and workload was graded ‘intermediate’ and only the last one related to the built environment, materials, and training was graded as ‘advanced.’ Additionally, major challenges were detected while filling in the WHO framework, linked mostly to the inadequate knowledge of the IPC focal person about the content of the tool and the length of time it took to respond to all the items on the questionnaire.

Previous studies conducted in Ghana and Uganda have reported similar findings. The study from Ghana using five of the WHO IPCAF core components found that 41.1% of the health facilities had a ‘basic’ level of compliance similar to what we found [17]. However, unlike LUH, 37.5% of these facilities in Ghana had senior level leaders participating in IPC activities and a dedicated budget was allocated [17]. In a study on implementation of IPC in health facilities in the Arua district of Uganda [18], 93.8% of these did not have an IPC committee, 93.8% reported an irregular supply of disposables and other supplies (such as gloves, soap, disinfectants), most facilities had no structures to monitor HCAI, and 72.6% never had in-service IPC training. However, the capacity to respond with adequate bed occupancy was present, which is similar to our findings. In contrast to LUH, a study from Germany revealed that 84.5% of the hospitals surveyed were in an ‘advanced’ level of IPC compliance [19]. While the overall compliance was good, multimodal strategies were graded ‘inadequate’ in the German study in line with our findings. The study from Germany also reported gaps in the components related to workload, staffing and bed occupancy, and built environment, materials, and equipment, where LUH performed better.

From a review in China, where 56 articles (including seven on hand hygiene and five on IPC education/training) qualified for data analysis, unlike LUH, where 98.1% of tertiary hospitals had an IPC committee, 67.2% had HAI surveillance, 85.8% had IPC guidelines, 75.5% provided post qualification IPC training, and 93.6% provided feedback on IPC performance indicators [20].

In a national survey conducted in Uganda in 2019, which included 42 health facilities including a national referral hospital, regional referral hospitals, general hospitals, private not for profit (PNFP) hospitals, private hospitals, and health centres. A total of 9.6% attained an advanced level, 52.4% had an intermediate level, 30.9% had a basic level like LUH, and 7.1% reached an inadequate level [21]. The overall average (433/800) level of compliance was graded ‘intermediate’, which was better than for LUH (Basic). Compared to LUH (basic), at least 60% of the health facilities had an ‘intermediate’ or better IPC level. Of the eight general hospitals, 37.5% had a ‘basic’ level like LUH. However, 37.5% had an ‘intermediate’ level and 12.5% had ‘advanced’ levels, which were better than LUH and 12.5% had a lower level (inadequate). In the five PNFP health facilities, 20% were at a ‘basic’ IPC level like LUH and the other 80% were at ‘intermediate’ (40%) and ‘advanced’ IPC levels. Unlike LUH, 78.5% had an IPC program, 71% had customized guidelines, 57% HCAI surveillance, 37% monitoring plan and targets, and 69% applied multimodal strategies. Like LUH, 50% had uninterrupted water, 67% adequate toilets, 67% adequate waste management supplies, and 93% adequate electricity supply, but, like LUH, 81% did not have an adequate supply of PPE and, in 40%, there was an unreliable supply of sterile and disposables items.

While testing the usability of WHO IPCAF in 46 countries, 52% of the respondents completed the tool in less than one hour [22], unlike, at LUH, where it took about two hours. This could be due to IPC trainings and exposure to the implementation in other countries, unlike LUH, which never had training and exposure to implementation. This implies that, with training and on-going IPC implementation, completing the tool becomes easier.

Our study had several strengths. We used a standardized tool recommended by the WHO to measure compliance to IPC policies and practices. Thus, the findings can be compared with those of other studies, which have used the same tool. This study provides a baseline assessment of IPC in LUH, which can be used to track progress when future assessments are done. We reported our study findings in line with STROBE (Strengthening the Reporting of Observational Studies in Epidemiology) guidelines. One limitation was that we conducted the study in a single hospital, and, thus, the findings have limited generalizability to other health care settings of Uganda.

Despite these limitations, the study has important implications. Based on the study findings, we make the following recommendations categorized into 3 groups: (i). “Easy to address recommendations”, which include: starting an IPC programme led by an IPC committee consisting of multidisciplinary professionals, designating one of the committee members as the IPC focal person with dedicated time to focus on IPC activities, constituting an IPC team consisting of full-time professionals trained to implement IPC activities, drawing an IPC action plan, which will guide the team to implement improvement practices, based on strengths and gaps, reviewing IPC activities periodically in executive meetings attended by senior level staff, putting in place IPC guidelines and adapting them to the local context, (ii)“Recommendations with moderate resource implications”, which include: instituting regular IPC monitoring, audit, and feedback, training all the health care providers, cleaners, and other personnel (such as administrative and managerial staff), patients and their family members in IPC practices utilizing in-house IPC expertise, putting in place a system of monitoring whether IPC practices are being implemented, providing feedback regularly to all the providers, providing adequate quantities of PPE and other disposable items, supplied in an uninterrupted manner since they are very vital even to prevent the spread of infections in the COVID-19 era, (iii) “Recommendations with major resource implications” which include: instituting HCAI surveillance, and putting in place a system for tracking the most important and relevant HCAIs in healthcare workers, e.g., tuberculosis, surgical site infections, hepatitis B, COVID-19, and *Clostridium difficile*, rolling out IPC guidelines and activities in other health facilities in Uganda to support IPC implementation, instituting follow-up assessments in future using the same tool in LUH and other health facilities so as to track the progress in implementing IPC policies and practice, and putting in place multimodal strategies to enhance IPC and allocating budgets for IPC activities.

## 5. Conclusions

In conclusion, the overall compliance to IPC in LUH in Uganda was not optimal. We identified serious gaps in multimodal strategies, supplies, and monitoring/surveillance. We have made specific recommendations for putting in place multimodal strategies and monitoring systems as well as for ensuring adequate and consistent supplies to address the gaps. The above recommendations include the drawing of an action plan, which must be implemented, with follow-up assessments using the same tool and feedback to track progress in LUH and other health facilities. We hope that such assessments will go a long way in improving IPC and in preventing HCAIs.

## Figures and Tables

**Table 1:** IPCAF scoring interpretation.

Score	Grading	Interpretation
0–200	Inadequate	IPC core component’s implementation is deficient. Significant improvement is required.
201–400	Basic	Some aspects of IPC core components are in place, but not sufficiently implemented. Further improvement required.
401–600	Intermediate	Most aspects of IPC core components are appropriately implemented. Continue to improve the scope and quality of implementation and focus on the development of long-term plans to sustain and further promote the existing programs.
601–800	Advanced	The IPC core components are fully implemented, according to the WHO recommendations, and appropriate to the needs of your facility.

**Table 2:** Baseline IPC compliance level of Lira University Hospital (November 2020).

IPCAF Core Components	Score *	Percentage	Interpretation
IPC programme	0.0	0.0	Inadequate
IPC guidelines	12.5	12.5	Inadequate
IPC education and training	35.0	35.0	Basic
Healthcare associated infection surveillance	25.0	25.0	Inadequate
Multimodal strategies	0.0	0.0	Inadequate
Monitoring/audit of IPC practices	0.0	0.0	Inadequate
Workload, staffing, andbed occupancy	70.0	70.0	Intermediate
Built environment, materials, and equipment for IPC	77.5	77.5	Advanced
Overall score (Maximum 800)	220	27.5	Basic

* Maximum score for each core component was 100. Component levels: 0–25% = inadequate; 25.1–50% = basic; 50.1–75% = intermediate; 75.1–100% = advanced. IPC = infection prevention and control. IPCAF = infection prevention and control action framework.

**Table 3:** Strengths and gaps in IPC (Infection prevention and control) programme, guidelines, IPC education and training identified during a baseline IPC assessment at Lira University Hospital, Uganda, in November 2020.

Components	Strengths	Gaps
**IPC programme**	None	No IPC programme No IPC team comprising full-time IPC professionals or their equivalent. Focal person does not have dedicated time for IPC activities. No IPC committee No clear commitment and support for IPC programme by senior leadership (no budget, not discussed in executive meetings) No microbiological laboratory support
**IPC Guidelines**	Facility guidelines for hand hygiene present Guideline in the facility consistent with national/international guidelines	No expertise for developing or adapting IPC guidelines There are no guidelines for: standard precautions, transmission-based precautions, outbreak management and preparedness, prevention of surgical site infections, prevention of vascular catheter-associated blood stream infections, prevention of hospital acquired pneumonia, prevention of catheter associated urinary tract infections, prevention of transmission of multidrug resistant (MDR) pathogens, disinfection and sterilization, healthcare worker protection and safety, injection safety, waste management and antibiotic stewardship Implementation of the guidelines not adapted according to the local needs and resources Healthcare workers have not received specific training related to new or updated IPC guidelines, which is not involved in implementation of IPC No regular monitoring of IPC implementation
**IPC Education and Training**	Personnel with IPC expertise to lead the training and additional non-IPC personnel with adequate skills to serve as trainers and mentors (link nurses or doctors, champions) present Some personnel trained using interactive training sessions (e.g., simulation, and bedside training) IPC training integrated in the clinical practice and training of some specialties (e.g., surgery)	Healthcare workers, cleaners, and other personnel directly involved in patient care have rarely received IPC training Administrative and managerial staff have never received IPC training No periodic evaluation of effectiveness of training programmes No specific IPC trainings for patients or family members No ongoing development/education offered for staff (e.g., regularly attending conferences, courses)

**Table 4:** Strengths and gaps in IPC (infection prevention and control) surveillance, monitoring, audit, and multimodal strategies observed during a baseline IPC assessment at Lira University Hospital, Uganda, in November 2020.

Components	Strengths	Gaps
**Healthcare Associated Infection Surveillance**	Personnel responsible for surveillance activities are present and trained in basic epidemiology, surveillance, and IPC (capacity to oversee surveillance methods, data management, and interpretation) Informatics/IT support to conduct surveillance available (e.g., equipment and electronic health records) Prioritization exercise has been done to determine the HCAIs to be targeted for surveillance according to the local context	No surveillance for surgical infections, device-associated infections (catheter associated UTI, central line associated blood stream infections, ventilator-associated pneumonia), clinically defined infections (definitions based only on clinical signs or symptoms in the absence of microbiological testing), infection caused by multi-drug resistant pathogens, local priority epidemic prone infections (norovirus, influenza, TB, SARS, Ebola, Lassa fever), infections in vulnerable populations (neonates, intensive care unit, immunocompromised, burn patients), infections that affect healthcare workers (Hep B or C, HIV, influenza) No regular evaluations to determine if surveillance is in line with the current needs and priorities for the facility **Methods for surveillance** No reliable surveillance case definitions according to international standards No standardized data collection methods No processes to regularly review data quality No adequate microbiology and laboratory capacity to support surveillance **Information analysis and dissemination** Surveillance data not used to make tailored facility-based plans for improving IPC Antimicrobial drug resistance not analysed on a regular basis Feedback of surveillance information not provided to frontline healthcare workers, clinical leaders/heads of departments, or senior management
**Multimodal Strategies**	None	Multimodal strategies not used to implement IPC interventions
**Monitoring/Audit of IPC Practices**	None	No trained personnel responsible for monitoring/audit of IPC practices and feedback No monitoring plan with clear goals, targets, and activities No processes and indicators monitored at the facility No annual survey using WHO Hand hygiene self-assessment framework No feedback report on the state of the IPC activities/performance No annual reporting of monitoring data No assessment of safety cultural factors in the facility

**Table 5:** Strengths and gaps in workload, staffing, bed occupancy, built environment, materials, and equipment for IPC (infection prevention and control) observed during a baseline IPC assessment at Lira University Hospital, Uganda, in November 2020.

Components	Strengths	Gaps
**Workload, Staffing, and Bed Occupancy**	Bed occupancy kept at one patient per bed No patients in facility placed in beds standing in the corridor outside of the room (including beds in the emergency department) Adequate spacing of >1 metre between patient beds ensured in the facility System in place to assess and respond when adequate bed capacity is exceeded or when staffing levels are low compared to needs Design of wards in facility in accordance with international standards regarding bed capacity in some departments	Staffing levels not assessed according to patient workload There is no agreed WHO or national ratio of healthcare workers to patients maintained across the facility
**Built Environment, Materials, and Equipment for IPC**	Water services available at all times and of sufficient quantities for all uses Reliable safe drinking water station present and accessible for staff, patients, and families at all times and in all locations/wards Functioning hand hygiene stations available at all points of care Sufficient energy/power available at day and night for all uses Functioning environmental ventilation available in patient care areas Have single patient rooms or rooms for cohorts of patients with similar pathogens if the number of isolation rooms is insufficient (TB, Measles, Ebola) Functional burial pit/fenced waste dump or municipal pick-up available for disposal of non-infectious general waste Incinerator or alternative treatment technology for treatment of infectious and sharp waste present, functional and of sufficient capacity Waste water treatment system present and functioning reliably Healthcare facility provides a dedicated decontamination area and/or sterile supply department for the decontamination and sterilization of medical devices and other items Reliably have sterile and disinfected equipment ready for use	Sufficient numbers of toilets or improved latrines available but not all are functioning for outpatient and inpatient settings No accessible record of cleaning, signed by the cleaners each day for surfaces or floors being cleaned Appropriate materials (buckets, mops, detergent) for cleaning available, but not well maintained PPE not continuously available in sufficient quantities Separate bins for waste collection available but inadequate: lids missing or bins more than ¾ full, only 2 bins (instead of 3), or bins at some but not all waste generation points Disposable items (gloves, injection safety devices) available when necessary but only sometimes

## Data Availability

All the data that concerns the content of this paper is already present in the paper.

## Supplementary Materials

The following are available online at https://www.mdpi.com/article/10.3390/tropicalmed6020069/s1. Table S1. Baseline assessment of infection prevention and control at the Lira University Hospital, Lira, Uganda in November 2020.

## References

[1] World Health Organisation Prevention of Hospital-Acquired Infections: A Practical Guide. http://apps.who.int/iris/bitstream/handle/10665/67350/WDO_CDS_CSR_EPH_2002.12.pdf.

[2] Allegranzi B., Nejad S.B., Combescure C., Graafmans W., Attar H., Donaldson L., Pittet D. (2011). Burden of endemic health-care-associated infection in developing countries: Systematic review and meta-analysis. Lancet.

[3] World Health Organization Keep Health Workers Safe to Keep Patients Safe. https://who.int/news/item/17-09-2020-keep-health-workers-safe-to-keep-patients-safe-who.

[4] Haque M., Sartelli M., McKimm J., Bakar M.A. (2018). Health care-associated infections—An overview. Infect. Drug Resist..

[5] Robert A. (2001). Weinstein Controlling Antimicrobial Resistance in Hospitals: Infection Control and Use of Antibiotics. Emerg. Infect. Dis..

[6] Kwok K.O., Read J.M., Tang A., Chen H., Riley S.K.K. (2018). A systematic review of transmission dynamic studies of methicillin-resistant Staphylococcus aureus in non-hospital residential facilities. BMC Infect. Dis..

[7] Labrague L.J., McEnroe-Petitte D.M., van de Mortel T., Nasirudeen A.M.A. (2018). A systematic review on hand hygiene knowledge and compliance in student nurses. Int. Nurs. Rev..

[8] Picheansanthian W., Chotibang J. (2015). Glove utilization in the prevention of cross transmission: A systematic review. JBI Database Syst. Rev. Implement Rep..

[9] Ofek Shlomai N., Rao S., Patole S. (2015). Efficacy of interventions to improve hand hygiene compliance in neonatal units: A systematic review and meta-analysis. Eur. J. Clin. Microbiol. Infect. Dis..

[10] Kock R., Becker K., Cookson B., van Gemert-Pijnen J.E., Harbarth S., Kluytmans J., Mielke M., Peters G., Skov R.L., Struelens M.J. (2014). Systematic literature analysis and review of targeted preventive measures to limit healthcare-associated infections by meticillin-resistant Staphylococcus aureus. Euro. Surveill..

[11] De Angelis G., Cataldo M.A., De Waure C., Venturiello S., La Torre G., Cauda R., Carmeli Y., Tacconelli E. (2014). Infection control and prevention measures to reduce the spread of vancomycin-resistant enterococci in hospitalized patients: A systematic review and meta-analysis. J. Antimicrob. Chemother..

[12] Murni I., Duke T., Triasih R., Kinney S., Daley A.J., Soenarto Y. (2013). Prevention of nosocomial infections in developing countries, a systematic review. Paediatr. Int. Child Health.

[13] Collins A.S., Hughes R.G. (2008). Chapter 41, Preventing Health Care-Associated Infections. Patient Safety and Quality.

[14] MoH Uganda MoH Uganda AMR National Action Plan. http://cddep.org/wp-content/uploads/2018/12/GoU_AMR-NAP.pdf.

[15] Allegranzi B., Kilpatrick C., Storr J., Kelley E., Park B.J., Donaldson L. (2017). Global Infection Prevention and Control Network. Global infection prevention and control priorities 2018-22: A call for action. Lancet Glob. Health.

[16] World Health Organization Infection Prevention and Control Assessment Framework at the Facility Level. https://www.who.int/infection-prevention/tools/core-components/IPCAF-facility.PDF.

[17] Oppong T.B., Amponsem-Boateng C., Kyere E.K.D., Wang Y., Gheisari Z., Oppong E.E., Opolot G., Duan G., Yang H. (2020). Infection Prevention and Control Preparedness Level and Associated Determinants in 56 Acute Healthcare Facilities in Ghana. Infect. Drug Resist..

[18] Wasswa P., Nalwadda C.K., Buregyeya E., Gitta S.N., Anguzu P., Nuwaha F. (2015). Implementation of Infection Control in Health Facilities in Arua District, Uganda: Cross Sectional Study. BMC Infect. Dis..

[19] Aghdassi S.J.S., Hansen S., Bischoff P., Behnke M., Gastmeier P. (2019). A national Survey on the Implementation of Key Infection Prevention and Control Structures in German Hospitals: Results from 736 Hospitals Conducting the WHO Infection Prevention and Control Assessment Framework (IPCAF). Antimicrob. Resist. Infect. Control..

[20] Wang J., Liu F., Tan J.B.X., Harbarth S., Pittet D., Zingg W. (2019). Implementation of Infection Prevention and Control in Acute Care Hospitals in Mainland China- a Systematic Review. Antimicrob. Resist. Infect. Control..

[21] Ministry of Health Uganda (2019). National Infection Prevention and Control Survey Report 2019.

[22] Tomczyk S., Agdhassi S., Storr J., Hansen S., Stewaedson A.J., Bischoff P., Gasmeier P., Allegranzi B. (2020). Testing of the WHO Infection Prevention and Control Assessment Framework at Acute Healthcare Facility. J. Hosp. Infect..

