# Supplementary material for: Infection Prevention and Control at Lira University Hospital, Uganda: More Needs to Be Done
Source: Trop Med Infect Dis. 2021 May 1;6(2):69. doi: 10.3390/tropicalmed6020069 (PMC8167580; doi:10.3390/tropicalmed6020069)
Supplement: Supplementary file 1 [file tropicalmed-06-00069-s001.zip › tropicalmed-1192778-supplementary.pdf]

Supplementary Table S1: Baseline assessment of infection prevention and control at the Lira University Hospital, Uganda, in November 2020

Core component 1: Infection Prevention and Control (IPC) programme

**Question**	**Answer**	**Score**
**1. Do you have an IPC programme?**		
Choose one answer	No√	0
	Yes, without clearly defined objectives	5
	Yes, with clearly defined objectives and annual activity plan	10
**2. Is the IPC programme supported by an IPC team comprising of IPC professionals?**		
Choose one answer	No √	0
	Not a team, *only* an IPC focal person	5
	Yes	10
**3. Does the IPC team have at least one full-time IPC professional or equivalent (nurse or doctor working 100% in IPC) available?**		
Choose one answer	No IPC professional available√	0
	No, *only* a part-time IPC professional available	2.5
	Yes, one per > 250 beds	5
	Yes, one per ≤ 250 beds	10
**4. Does the IPC team or focal person have dedicated time for IPC activities?**		
	No √	0
	Yes	10
**5. Does the IPC team include both doctors and nurses?**		
	No √	0
	Yes	10
**6. Do you have an IPC committee actively supporting the IPC team?**		
	No √	0
	Yes	10
**7. Are any of the following professional groups represented/ included in the IPC committee?**		
Senior facility leadership (for example, administrative director,	No √<br>Yes	0<br>5

chief executive officer [CEO], medical director)		
Senior clinical staff (for example, physician, nurse)	No √<br>Yes	0<br>2.5
Facility management (for example, biosafety, waste, and those tasked with addressing water, sanitation, and hygiene [WASH])	No √<br>Yes	0<br>2.5
**8. Do you have clearly defined IPC objectives (that is, in specific critical areas)?** Choose one answer		
	No√	0
	Yes, IPC objectives *only*	2.5
	Yes, IPC objectives and measurable outcome indicators (that is, adequate measures for improvement)	5
	Yes, IPC objectives, measurable outcome indicators and set future targets	10
**9. Does the senior facility leadership show clear commitment and support for the IPC programme:**		
By an allocated budget specifically for the IPC programme (that is, covering IPC activities, including salaries)?	No √<br>Yes	0<br>5
By demonstrable support for IPC objectives and indicators within the facility (for example, at executive level meetings, executive rounds, participation in morbidity and mortality meetings)?	No √<br>Yes	0<br>5
**10. Does your facility have microbiological laboratory support (either present on or off site) for routine day-to-day use?** Choose one answer		
	No √	0
	Yes, but not delivering results reliably (timely and of sufficient quality)	5
	Yes, and delivering results reliably (timely and of sufficient	10

	quality)	
**Subtotal score**		**00/100**

Core component 2: Infection Prevention and Control (IPC) Guidelines

**Question**	**Answer**	**Score**
**1. Does your facility have the expertise (in IPC and/or infectious diseases) for developing or adapting guidelines?**		
	No √	0
	Yes	7.5
**2. Does your facility have guidelines available for:**		
Guidelines for standard precautions?	No √	0
	Yes	2.5
Guidelines Hand hygiene?	No	0
	Yes √	2.5
Guidelines Transmission-based precautions?	No √	0
	Yes	2.5
Guidelines Outbreak management and preparedness?	No√	0
	Yes	2.5
Prevention of surgical site infection?	No √	0
	Yes	2.5
Prevention of vascular catheter-associated bloodstream infections?	No √	0
	Yes	2.5
Prevention of hospital-acquired pneumonia ([HAP]; all types of HAP, including (but not exclusively) ventilator-associated pneumonia)?	No √	0
	Yes	2.5
Prevention of catheter-associated urinary tract infections?	No√	0
	Yes	2.5
Prevention of transmission of multidrug-resistant (MDR) pathogens?	No √	0
	Yes	2.5
Disinfection and sterilization?	No √	0
	Yes	2.5
Health care worker protection and safety	No√	0
	Yes	2.5

Injection safety?	No √	0
	Yes	2.5
Waste management?	No √	0
	Yes	2.5
Antibiotic stewardship?	No √	0
	Yes	2.5
**3. Are the guidelines in your facility consistent with national/international guidelines (if they exist)?**	No<br>Yes √	0<br>10
**4. Is implementation of the guidelines adapted**10 **according to the local needs and resources while maintaining key IPC standards?**	No√<br>Yes	0<br>10
**5. Are frontline health care workers involved in both planning and executing the implementation of IPC guidelines in addition to IPC personnel?**	No√<br>Yes	0<br>10
**6. Are relevant stakeholders (for example, lead doctors and nurses, hospital managers, quality management) involved in the development and adaptation of the IPC guidelines in addition to IPC personnel?**	No√<br>Yes	0<br>7.5
**7. Do health care workers receive specific training related to new or updated IPC guidelines introduced in the facility?**	No√<br>Yes	0<br>10
**8.Do you regularly monitor the implementation of at least some of the IPC guidelines in your facility?**	No√<br>Yes	0<br>10
**Subtotal score**		**12.5/100**

Core component 3: Infection Prevention and Control (IPC) education and training

**Question**	**Answer**	**Score**
**1. Are there personnel with the IPC expertise (in IPC and/or infectious diseases) to lead IPC training?**	No<br>Yes √	0<br>10
**2. Are there additional non-IPC personnel with adequate skills to serve as trainers and mentors (for example, link nurses or doctors, champions)?** Chooseoneanswer	No<br>Yes √	0<br>10
**3. How frequently do health care workers receive training regarding IPC in your facility?** Chooseoneanswer	Never or rarely √	0

	New employee orientation *only* for health care workers	5
	New employee orientation and regular (at least annually) IPC training for health care workers offered but not mandatory	10
	New employee orientation and regular (at least annually) mandatory IPC training for all health care workers	15
**4.How frequently do cleaners and other personnel directly involved in patient care receive training regarding IPC in your facility?** Chooseoneanswer	Never or rarely √	0
	New employee orientation *only* for other personnel	5
	New employee orientation and regular (at least annually) training for other personnel offered but not mandatory	10
	New employee orientation and regular (at least annually) mandatory IPC training for other personnel	15
**5.Does administrative and managerial staff receive general training regarding IPC in your facility?**	No √<br>Yes	0<br>5
**6. How are health care workers and other personnel trained?** Chooseoneanswer	No training available	0
	Using written information and/or oral instruction and/or e-learning *only*	5
	Includes *additional* interactive training sessions (for example, simulation and/or bedside training) √	10
**7.Are there periodic evaluations of the effectiveness of training programmes (for example, hand hygiene**	No √<br>Yes, but not regularly	0<br>5

**audits, other checks on knowledge)?**<br>Chooseoneanswer	Yes, regularly (at least annually)	10
**8. Is IPC training integrated in the clinical practice and training of other specialties (for example, training of surgeons involves aspects of IPC)?**<br>Chooseoneanswer	No<br>Yes, in some disciplines√<br>Yes, in all disciplines	0<br>5<br>10
**9. Is there specific IPC training for patients or family membersto minimize the potential for health care-associated infections(for example, immunosuppressed patients, patients with invasivedevices, patients with multidrug-resistant infections)?**	No √	0
	Yes	5
**10. Is ongoing development/education offered for IPC staff (for example, by regularly attending conferences, courses)?**	No √<br>Yes	0<br>10
**Subtotal score**		**35/100**

Core component 4: Health care-associated infection (HAI) surveillance

Question	Answer	Score
Organization of surveillance		
**1. Is surveillance a defined component of your IPC programme?**	No<br>Yes√	0<br>5
**2. Do you have personnel responsible for surveillance activities?**	No<br>Yes√	0<br>5
**3. Have the professionals responsible for surveillance activities been trained in basic epidemiology, surveillance and IPC (that is, capacity to oversee surveillance methods, data management and interpretation)?**	No<br>Yes√	0<br>5
**4.Do you have informatics/IT support to conduct your surveillance (for example, equipment, mobile technologies, electronic health records)?**	No<br>Yes√	0<br>5
Priorities for surveillance-defined according to the scope of care		
**5. Do you go through a prioritization exercise to determine the HAIs to be targeted for surveillance according to the local context (that is, identifying infections that are major causes of morbidity and mortality in the facility)?**	No<br>Yes√	0<br>5
**6.In your facility is surveillance conducted for**		
Surveillance conducted for Surgical site infections?	No √<br>Yes	0<br>2.5
Surveillance conducted for Device-associated infections (for example, catheter-associated urinary tract infections, central line-associated bloodstream infections, peripheral-line associated bloodstream infections, ventilator-associated pneumonia)?	No √<br>Yes	0<br>2.5
Surveillance conducted for Clinically-defined infections (for example, definitions based only on clinical signs or symptoms in the absence of microbiological testing)?	No√<br>Yes	0<br>2.5
Surveillance conducted for Colonization or infections caused by multidrug-resistant13 pathogens according to your local epidemiological situation?	No √<br>Yes	0<br>2.5
Surveillance conducted for Local priority epidemic-prone infections (for example, norovirus, influenza, tuberculosis [TB], severe acute respiratory syndrome [SARS], Ebola, Lassa fever)?	No √<br>Yes	0<br>2.5
Surveillance conducted for Infections in vulnerable populations (for example, neonates, intensive care unit, immunocompromised, burn patients)?	No √<br>Yes	0<br>2.5

Surveillance conducted for Infections that may affect health care workers in clinical, laboratory, or other settings (for example, hepatitis B or C, human immunodeficiency virus [HIV], influenza)?	No √<br>Yes	0<br>2.5
**7. Do you regularly evaluate if your surveillance is in line with the current needs and priorities of your facility?**	No √<br>Yes	0<br>5
Methods of surveillance		
**8. Do you use reliable surveillance case definitions (defined numerator and denominator according to international definitions [e.g. CDC NHSN/ ECDC] or if adapted, through an evidence-based adaptation process and expert consultation?**	No √<br>Yes	0<br>5
**9. Do you use standardized data collection methods (for example, active prospective surveillance) according to internationalsurveillance protocols (for example, CDC NHSN/ECDC) or ifadapted, through an evidence-based adaptation process and expert consultation?**	No √<br>Yes	0<br>5
**10. Do you have processes in place to regularly review data quality (for example, assessment of case report forms, review of microbiology results, denominator determination, etc.)?**	No √<br>Yes	0<br>5

**11. Do you have adequate microbiology and laboratory capacity to support surveillance?**		
**Choose one answer**	No √	0
	Yes, can differentiate gram-positive/negative strains but cannot identify pathogens	2.5
	Yes,can reliably identify pathogens (for example, isolate identification) in a timely manner	5
	Yes,can reliably identify pathogens and antimicrobial drug resistance patterns (this is, susceptibilities) in a timely manner	10

Information analysis and dissemination/data use, linkage, and governance		
**12. Are surveillance data used to make tailored unit/facility-based plans for the improvement of IPC practices?**	No √<br>Yes	0<br>5
**13. Do you analyze antimicrobial drug resistance on a regular basis (for example, quarterly/half-yearly/annually)?**	No √<br>Yes	0<br>5
14. **Do you regularly (for example, quarterly/half-yearly/annually) feedback up-to-date surveillance information to:**		
Frontline health care workers (doctors/nurses)?	No √	0

	Yes	2.5
Clinical leaders/heads of department	No √<br>Yes	0<br>2.5
IPC committee	No √<br>Yes	0<br>2.5
Non-clinical management/administration (chief executive officer/chief financial officer)?	No √<br>Yes	0<br>2.5
**15. How do you feedback up-to-date surveillance information? (at least annually)**		
**Choose one answer**	No feedback √	0
	By written/oral information only	2.5
	By presentation and interactive problem-orientated solution finding	7.5
**Subtotal score**		**25/100**

□

□

Core component 5: Multimodal strategies16 for implementation of infection prevention and control (IPC) interventions

Question		Answer	Score
**1. Do you use multimodal strategies to implement IPC interventions?**		No √<br>Yes	0<br>15
**2. Do your multimodal strategies include any or all of the following elements:**<br>*Choose **ONE** answer (the most accurate) per element*			
**System change**	Element not included in multimodal strategies		0
	Interventions to ensure the necessary infrastructure and continuous availability of supplies are in place		5
	Interventions to ensure the necessary infrastructure and continuous availability of supplies are in place and addressing ergonomics17 and accessibility, such as the best placement of central venous catheter set and tray		10
**Education and training**	Element not included in multimodal strategies √		0
	Written information and/or oral instruction and/or e-learning *only*		5
	Additional interactive training sessions (includes simulation and/or bedsidetraining)		10
**Monitoring and feedback**	Element not included in multimodal strategies √		0
	Monitoring compliance with process or outcome indicators (for example,		5
	Monitoring compliance and providing timely feedback of monitoring results to health care workers and key players		10
**Communications and reminders**	Element not included in multimodal strategies √		0
	Reminders, posters, or other advocacy/awareness-raising tools to promotethe intervention		5
	Additional methods/initiatives to improve team communication across unitsand disciplines (for example, by establishing regular case conferences andfeedback rounds)		10
**Safety climate and culture change**	Element not included in multimodal strategies √		0
	Managers/leaders show visible support and act as champions and role models, promoting an adaptive approach18 and strengthening a culture thatsupports IPC, patient safety and quality		5

	Additionally, teams and individuals are empowered so that they perceiveownership of the intervention (for example, by participatory feedback rounds)		10
**3. Is a multidisciplinary team used to implement IPC multimodal strategies?**		No √	0
		Yes	15
**4.Do you regularly link to colleagues from qualityimprovement and patient safety to develop andpromote IPC multimodal strategies?**		No √<br>Yes	0<br>10
**5.Do these strategies include bundles or checklists?**		No √	0
		Yes	10
**Subtotal score**			**00/100**

Core component 6: Monitoring/audit of IPC practices and feedback

Question	Answer	Score
**1. Do you have trained personnel responsible for monitoring/audit of IPC practices and feedback?**		
	No √	0
	Yes	10
**2. Do you have a well-defined monitoring plan with clear goals, targets and activities (including tools to collect data in a systematic way)?**		
	No √	0
	Yes	7.5
**3. Which processes and indicators do you monitor in your facility?** Tick all that apply		
	None √	0
	Hand hygiene compliance (using the WHO hand hygiene observation tool20 or equivalent)	5
	Intravascular catheter insertion and/or care	5
	Wound dressing change	5
	Transmission-based precautions and isolation to prevent the spread of multidrug resistant organisms (MDRO)	5
	Cleaning of the ward environment	5
	Disinfection and sterilization of medical equipment/instruments	5
	Consumption/usage of alcohol-based handrub or soap	5
	Consumption/usage of antimicrobial agents	5
	Waste management	5
**4. How frequently is the *WHO Hand Hygiene Self-AssessmentFramework Survey* undertaken?**		
Chooseoneanswer	Never √	0
	Periodically, but no regular schedule	2.5
	At leastannually	5
**Do you feedback auditing reports (for example, feedback on handhygiene compliance data or other processes) on the state of the IPCactivities/performance?**		
**5.** Tick all that apply	No reporting √	0
	Yes, within the IPC team	2.5

	Yes, to department leaders and managers in the areas being audited	2.5
	Yes, to frontline health care workers	2.5
	Yes, to the IPC committee or quality of care committees or equivalent	2.5
	Yes, to hospital management and senior administration	2.5
**6. Is the reporting of monitoring data undertaken regularly (at least annually)?**	No √	0
	Yes	10
**7. Are monitoring and feedback of IPC processes and indicatorsperformed in a "blame-free" institutional culture aimed atimprovement and behavioural change?**	No √	0
	Yes	5
**8. Do you assess safety cultural factors in your facility (for example, by using other surveys such as HSOPSC, SAQ, PSCHO, HSC22)**	No √<br>Yes	0<br>5
**Subtotal score**		00/100

Core component 7: Workload, staffing and bed

**Question**	**Answer**	**Score**
Staffing		
**1. Are appropriate staffing levels assessed in your facility accordingto patient workload using national standards or a standard staffingneeds assessment tool such as the** ***WHO Workload indicators ofstaffing need*****24method?**	No √	0
	Yes	5
**2. Is an agreed (that is, WHO or national) ratio of health care workersto patientsmaintained across your facility?** Chooseoneanswer	No √	0
	Yes, for staff in less than 50% of units	5
	Yes, for staff in more than 50% of units	10
	Yes, for all health care workers in the facility	15
**3. Is a system in place in your facility to act on the results of thestaffing needs assessments when staffing levels are**	No	0
	Yes √	10

**deemed to betoo low?**		
Bed occupancy		
**4. Is the design of wards in your facility in accordance withinternational standards**26 **regarding bed capacity?** Chooseoneanswer	No √	0
	Yes but only in certain departments	5
	Yes, for all departments (including emergency department and pediatrics)	15
**5. Is bed occupancy in your facility kept to one patient per bed?** Chooseoneanswer	No	0
	Yes, but *only* in certain departments	5
	Yes, for all units (including emergency departments and pediatrics) √	15
**6. Are patients in your facility placed in beds standing in the corridoroutside of the room (including beds in the emergency department)?** Chooseoneanswer	Yes, more frequently than twice a week	0
	Yes, less frequently than twice a week	5
	No √	15
**7. Is adequate spacing of > 1 meter between patient beds ensuredin your facility?** Chooseoneanswer	No	0
	Yes, but only in certain departments	5
	Yes, for all departments (including emergency department and pediatrics) √	15
**8. Is a system in place in your facility to assess and respond when adequate bed capacity is exceeded?** Chooseoneanswer	No	0
	Yes, this is the responsibility of the head of department	5
	Yes, this is the responsibility of the hospital administration/ management√	10
**Subtotal score**		70/**100**

Core component 8: Built environment, materials and equipment for IPC at the facility level

Question	Answer	Score
Water		
**1. Are water services available at all times and of sufficient quantityfor all uses (for example, hand washing, drinking, personal hygiene, medical activities, sterilization, decontamination, cleaning andlaundry)?** Chooseoneanswer	No, available on average < 5 days per week	0
	Yes, available on average ≥ 5 days per week or every day but not of sufficient quantity	2.5
	Yes, every day and of sufficient quantity √	7.5
**2. Is a reliable safe drinking water station present and accessible for staffs, patients and families at all times and in all locations/wards?** Chooseoneanswer	No, notavailable	0
	Sometimes, or only in some places or not available for all users	2.5
	Yes, accessible at all times and for all wards/groups √	7.5
Hand hygiene and sanitation facilities		
**3. Are functioning hand hygiene stations (that is, alcohol-basedhandrub solution or soap and water and clean single-use towels)available at all points of care?** Chooseoneanswer	No, not present	0
	Yes, stations present, but supplies are not reliably available	2.5
	Yes, with reliably available supplies √	7.5
**4. In your facility, are ≥ 4 toilets or improved latrines available foroutpatient settings or ≥ 1 per 20 users for inpatient settings?** Chooseoneanswer	Less than required number of toilets or latrines available and functioning	0
	Sufficient number present but not all functioning √	2.5
	Sufficient number present and functioning	7.5
Power supply, ventilation and cleaning		
**5. In your health care facility, is sufficient energy/power supplyboiling water, sterilization and decontamination, incineration oralternative treatment technologies, electronic medical devices,general lighting of areas where health care procedures are performedto ensure safe provision of health care and lighting of toilet facilitiesand showers)?** Chooseoneanswer	No	0
	Yes, sometimes or only in some of the mentioned areas	2.5
	Yes, always and in all mentioned areas√	5

**6. Is functioning environmental ventilation (natural or mechanical) available in patient care areas?**	No	0
	Yes √	5
**7. For floors and horizontal work surfaces, is there an accessiblerecord of cleaning, signed by the cleaners each day?** Chooseoneanswer	No record of floors and surfaces being cleaned √	0
	Record exists, but is not completed and signed daily or is outdated	2.5
	Yes, record completed and signed daily	5
**8. Are appropriate and well-maintained materials for cleaning (for example, detergent, mops, buckets, etc.) available?** Chooseoneanswer	No materialsavailable	0
	Yes, available, but not well maintained√	2.5
	Yes, available and well-maintained	5
**Patient placement and personal protective equipment (PPE) in health care settings**		
**9. Do you have single patient rooms or rooms for cohorting patients with similar pathogens if the number of isolation rooms is insufficient (for example, TB, measles, cholera, Ebola, SARS)?** Chooseoneanswer	No	0
	No single rooms but rather rooms suitable for patient cohorting available<br>Yes, single rooms are available √	2.5<br>7.5
**10. Is PPE available at all times and in sufficient quantity for alluses for all health care workers?** Chooseoneanswer	No<br>Yes, but not continuously available in sufficient quantities √<br>Yes, continuouslyavailable in sufficientquantities	0<br>2.5<br>7.5
**Medical waste management and sewage**		
**11. Do you have functional waste collection containers for non-infectious (general) waste, infectious waste and, sharps waste in close proximity to all waste generation points?** Chooseoneanswer	No bins or separate sharps disposal	0
	Separate bins present but lids missing more than ¾ full; only two bins (instead of three); or bins at some but not all waste generation points √	2.5
	Yes	5
**12. Is a functional burial pit/fenced waste dump or municipal pick-up available for disposal of non-infectious (non-hazardous/general waste)?** Chooseoneanswer	No pit or other disposal method used	0
	Pit in facility but insufficient dimensions; pits/dumps overfilled or not fenced/locked; or irregular municipal waste pick up	2.5
	Yes √	5
**13. Is an incinerator or alternative**	No, none present	0

**treatment technology for the treatment of infectious and sharp waste (for example, an autoclave) present (either present on or off site and operated by a licensed waste management service), functional and of a sufficient capacity?**<br>Chooseoneanswer	Present, but notfunctional	1
	Yes √	5
**14. Is a wastewater treatment system (for example, septic tank followed by drainage pit) present (either on or off site) and functioning reliably?**<br>Chooseoneanswer	No, not present	0
	Yes, but not functioning reliably	2.5
	Yes and functioning reliably √	5

Decontaminationandsterilization		
**15. Does your health care facility provide a dedicated decontamination area and/or sterile supply department (either present on or off site and operated by a licensed decontamination management service) for the decontamination and sterilization of medical devices and other items/equipment?**<br>Chooseoneanswer	No, not present	0
	Yes, but not functioning reliably	2.5
	Yes and functioning reliably √	5
**16. Do you reliably have sterile and disinfected equipment ready for use?**<br>Chooseoneanswer	No, available on average < five days per week	0
	Yes, available on average ≥ five days per week or every day, but not sufficient quantity	2.5
	Yes, available every day and of sufficient quantity √	5
**17. Are disposable items available when necessary? (for example, injection safety**	No, notavailable	0
	Yes, but only sometimes available √	2.5

**devices, examination gloves)**<br>Chooseoneanswer	Yes, continuouslyavailable	5
**Subtotal score**		77.5/**100**

Time at the end of administering the questions……………………….
